# Reviewers acknowledgement

**DOI:** 10.1002/2211-5463.13045

**Published:** 2020-12-03

The editors of *FEBS Open Bio* would like to thank all those who have given their time and expertise to review articles submitted for publication in Volume 10. Although *FEBS Open Bio* does not seek to judge the importance of submissions, our reviewers carefully scrutinize the experimental design and results of all papers and challenge authors about their conclusions.

The names of these reviewers are listed below; we apologize if we have inadvertently omitted anyone.

Maolake Aerken

Jon Agirre

Yukio Ago

Francisco Aguayo

Aamir Ahmad

Moaz Ahmad

Wado Akamatsu

Gul Akdogan

S. Amanda Ali

Chiara Allegretti

Fernando Almeida‐Souza

Sergio Alonso

Noelia Alonso Gonzalez

Peter Alston

Valentin Ambroise

Yu An

Xu An

Eduardo Andrés‐León

Oleh Andrukhov

Ileana Antony‐Debre

Arnoldo Aquino‐Gálvez

Paolo Arosio

Yaw Asare

Avraham Ashkenazi

Essam Assali

Ianire Astobiza

Nese Atabey

Rosario Avolio

Raheela Awais

Ester Badami

Lina Badimon

Leila Bagheri

Monika Baj‐Krzyworzeka

Oliver Bajt

H. Scott Baldwin

Marcella Barbarino

Benoit Barbeau

Jose Alexandre Marzagão Barbuto

Elias Barquero

Niall Barron

Riyaz Basha

Reetobrata Basu

Reinhard Bauer

Jose Belizário

Stephen G. Bell

Karim Benabdellah

Manju Benakanakere

Alexandre Benedetto

Nir Ben‐Tal

Subrat Bhattamisra

Vikrant K. Bhosle

Marco Binder

Thomas Binz

Tomasz Blicharski

Arnold Bolomsky

Usa Boonyuen

Francesca Bosisio

Veronique Braud

Gustavo Bressan

Mariarosaria Bucci

Chloe Bulinski

Peter Burkovics

Verónica A. Burzio

Bernadette Byrne

Jonas Bystrom

Vittorio Calabrese

Vincenzo Calvanese

Laura Calvo

Simon Camilleri

Johnathan Canton

Xinsheng Cao

Hui‐Ling Cao

Enrico Capobianco

Maria Francesca Cardone

Inês Antunes Cardoso Pereira

Christopher P. Cardozo

Daniela Carlisi

Ingrid Carvacho

José Carvalheira

Gustavo Carvalho Dias

Claudia Cava

Denise Cavalcanti

Federica Censi

Young Chan Chae

Jiani Chai

Anupam Chakravarty

Te‐Sheng Chang

Kuo‐Wei Chang

Kang Chanhee

Rosamund Chapman

Steve Charette

Ashutosh Chauhan

Gabriela Chavarria

Esteban Chaves

Wei Chen

Qilong Chen

Gang Chen

Yan Chen

Wei‐Yu Chen

Liyang Chen

Jian‐ye Chen

Xiaohua Chen

Qi Chen

Guang Chen

Shiyuan Cheng

Xuanhong Cheng

Peter P. Cherepanov

I. Tsang Chiang

Agata Chmurzynska

David W. Christianson

Jun Chu

Michelle Clark

Nicholas Clemons

Vicki Clifton

Randall J. Cohrs

Alessandra Colciago

Patricia Coltri

Nicholas Cosford

Bruno Costa‐Silva

Xenia Crespo

Francisco Javier Cubero

Martha S. Cyert

Zsolt Czimmerer

Michal Dabrowski

André Damasio

Christoph Daniel

Matthew Daniels

Jean Luc Darlix

Parimal Das

Zbigniew Dauter

John Davies

Marcos Roberto de Oliveira

Alessandro Melo Deana

Liesbeth Demuyser

Hansong Deng

Mingming Deng

Jayne Dennis

Carsten Deppermann

Lalit Deshmukh

Kishor Devalaraja‐Narashimha

Sandra Dias

Paula Diaz

Antonio J. Diaz‐Quintana

Luciano DiTacchio

Ian M.C. Dixon

Angela Dona

Hai‐Cao Dong

Gabriella Dorazi

Leonardo dos Reis Silveira

Alfonso Dueñas

Didrik Dukefoss

Nicolas Dumaz

M. Beatriz Durán‐Alonso

Helene Dutartre

Dibyendu Dutta

Taisuke Ekino

Rihan El Bezawy

Abd El Samad

Selvakumar Elangovan

Atsuki En

Patrick England

Guochang Fan

Zhipeng Fan

Jing Fang

Wissam Faour

Macarena Farcuh

Ahmad Fari

Injie Omar Fawzy

Dennis Liang Fei

Emanuela Felley‐Bosco

Yansheng Feng

Alejandro Ferrando

Jarlei Fiamoncini

Tiago Figueira

Susetta Finotto

Christopher Fitzpatrick

Matthew Fogarty

José Claudio Fonseca Moreira

Federico Forneris

Florentia Fostira

Philippe Frachet

Hector Franco

Richard Frank

Marie Frazer

Andrew Fry

Qiantang Fu

Liyun Fu

Jing Fu

Ko Fujimori

Atsunori Fukuhara

Clemens Furnes

Mattheu Gagnon

Sanjeev Anant Galande

Assimina Galli‐Tsinopoulou

Pamela Gan

José Manuel García‐Heredia

Fabrizio Gardoni

James A. Garnett

Galia Gat‐Yablonski

Damian Gaweł

Anne George

Fabiana Geraci

David Gerrard

Sergio Giannattasio

Gianluca Giavarese

Tobias Giessen

Ferhan Girgin Sağın

Dasantila Golemi‐Kotra

Pablo Gonzalez

Aleksander Grabiec

Sergio A. Gradilone

Karol Granados

Giuseppe Grasso

Darrell Green

Jonathan Green

Jenna Gregory

Ramon Grima

Wolfram Gronwald

Valentina Grossi

Paolo Grumati

Xinbin Gu

De‐Leung Gu

Chen Gu

Alejandra Guerra‐Castellano

Roberto Guidetti

James Gumbart

Qingyuan Guo

Da‐Long Guo

Bhawna Gupta

Carino Gurjao

Oleg Gusev

Caterina Guzman Verri

Ana Gvozdenovic

R. Haddad

Kamyar Hadian

Nguyen Hai Dang

George Hajishengallis

Barry Halliwell

Rikinari Hanayama

Sumit Handa

Christopher Hano

Jennifer Hardingham

K.B. Harikumar

Brittney Harrington

Narumi Hashikawa‐Hobara

Kenji Hashimoto

Ari Hashimoto

Hidetoshi Hayashi

Yong He

Wei He

Reinhard Hehl

Michael Helms

Simon Hennig

Kyun Heo

Katharine Herbert

Pim Hermans

Andrew Higham

Kevin Hildebrand

Kouji Hirota

Joseph Ho

Nathan Hodson

Federico Hoffmann

John D. Hooper

Jue Hou

Guy A. Howard

Chia‐Ling Hsieh

Yen‐Ping Hsueh

Jia Hu

Shijun Hu

Kun Huang

Yongye Huang

Pablo Huertas

Jae Hur

Maik Huttemann

Siaw San Hwang

Michael Ibba

Koichi Iijima

Svenja Illien‐Junger

Stephan Immenschuh

Rie Ishikawa

Shoichi Ishiura

Taichi Isobe

Eisuke Itakura

Kazuhisa Iwabuchi

Yasuhiko Izumi

Hieronim Jabubowski

Jeffrey Jacobson

Aaron W. James

Elisa Helena Farias Jandrey

Xin‐Ying Ji

Yanfang Jiang

Bin Jiang

Eijiro Jimi

Haiming Jin

Rongsheng Jin

Behrooz Johari

Philip E. Johnson

Kenneth A. Johnson

Cameron Johnstone

Marie Joossens

Joana Jorge

Alexander Kalachev

Bernadett Kalmar

Ryutaro Kamijo

Atsushi Kamiya

Masato Kanazawa

Joanna Kania

Mukesh Kapoor

Fatah Kashanchi

Muzaffer Ahmad Kassab

Naoki Katase

Hironori Katoh

Yukio Kawahara

Kazumichi Kawakubo

Keiko Kawauchi

Younghoon Kee

Andrew Kelleher

Eric E. Kelley

Thomas Kenslar

Mona Khafaji

Bilon Khambu

Iman Aftab Khan

KumKum Khanna

Choy Yuen Khew

Khatereh Khorsandi

Say‐June Kim

Yunhee Kim

Seok‐Jun Kim

Sachie Kimura

K. Klempnauer

Jakob G. Knudsen

Yoshihiro Kobashigawa

Makoto Kobayashi

Yuta Kohro

Yu‐Ichiro Koma

Eisaku Kondo

Min Kong

Adam Konopka

Neeltje Kootstra

Janko Kos

Ioannis Kourtzelis

Olha Koval

Mitsumasa Koyanagi

Akiko Kozaki

Heinrich Kroukamp

Vladimir Krystof

Tomohiko Kubo

Takashi Kudo

Veerapol Kukongviriyapun

Arvind Kumar

Sandeep Kumar

Sarawut Kumphune

Ozgur Kutuk

Marie Kveiborg

So Hee Kwon

Lawrence N. Kwong

Verena Labi

Leticia Labriola

Daniel Lajeunesse

Sowmya Lakshmi

Yu‐Yu Lan

Janine M. LaSalle

Gregory Lavieu

Nicole LeCorre

Kang‐Hoon Lee

Daekee Lee

Tzong‐Shyuan Lee

S.Y. Lee

Xiujuan Lei

Zhao Lei

Shi Lei

Andreia Machado Leopoldino

Martina Lepore Signorile

Aaron Lerner

François Letourneur

Valeria Levi

Bodo Levkau

Mingchuan Li

Yigang Li

Shenglong Li

Chunlai Li

Shugang Li

Po‐Huang Liang

Florence Lichou

Daniel Lietha

Ya Chee Lim

Chieh‐Yu Lin

Chin‐Yo Lin

Cho‐hao Lin

Ya‐Wen Lin

Angus J.C. Lindsay

Mikael Lindström

Maxine Linial

Dirk Linke

Zhenguo Liu

Jianwei Liu

Xuxiang Liu

Rui Liu

Ruijiang Liu

Shu‐Lai Liu

César Lopez‐Camarillo

O. Lorenzo

Péter Lőrincz

Jesus Losada

Diana Lousa

Qingchun Lu

Hui‐Ping Lu

Lingeng Lu

Jin‐Jian Lu

Giuseppe Lucarelli

Agnieszka Ludwikow

Yongwen Luo

G.W. Gant Luxton

Fu‐chao Ma

Wei Ma

Sihui Ma

Andrew MacLean

Yasuhiro Maejima

Maria Magdalena Barreca

Kiran Mahajan

Muhammad Faraz Arshad Malik

Jose J.G. Marin

Luis Antonio Mariñas‐Pardo

Mozart Marins

Javier Martinez

Jonathan Martinez‐Fabregas

Sabata Martino

Simona Martinotti

Pedro Matias

Pedro Matos

Shu‐ichi Matsuzawa

Konstantinos Mavridis

A. John McKinnon

Rafael Medina

Lucia Melguizo‐Rodríguez

Gina Mendez‐Callejas

Luis Menendez‐Arias

Dora K. Menyhard

Michael Menze

Stefano Menzo

Francesca Marino Merlo

Akira Mima

Hamid Reza Mirzaei

Aleksandra Misicka

Tomohiro Mizobata

Bushra Moiz

Pooi Ling Mok

Nurbubu Tentievna Moldogazieva

Antonio Molina Fernández

Eszter Molnar

Timothy Moore

Mohammad Amin Moosavi

Joanna Moraczewska

Gary Moran

Randy Morgenstein

Taisuke Mori

Yoshinori Moriyama

Igor Morozov

Ken Motohashi

Jorge Mukdsi

Manishi Mukesh

Saikat Mukhopadhyay

Stefan Muller

Michael J. Mulvany

Shona Murphy

Julian Mustroph

Parisa Naeli

Előd Ernő Nagy

Peter Nagy

Javid Nahand

Firzan Nainu

So Nakagawa

Hitoshi Nakamoto

Masayoshi Nakamura

Hajime Nakao

Ikuhiko Nakase

Takanobu Nakazawa

Pratima Nangia‐Makker

Ophir Nave

Hovav Nechushtan

Wataru Nemoto

V.V. Nenasheva

Jennifer Ngo

Marc Nicklaus

R. Henrik Nilsson

Fusanori Nishimura

Hiroshi Nishina

Hiroshi Nomura

Saori Nonaka

Masafumi Nozawa

Fena Ochs

Toshiaki Ohara

Chio Oka

Shigeru Okada

Yohei Okada

Valentyn Oksenych

Rodrigo Cardoso de Oliveira

Gustavo Olivos‐Ramirez

Ana O'Loghlen

Hiroshi Omote

Daisuke Ono

Yasuhito Onodera

Karariina Oorni

Miguel O'Rayan

Darren O'Rielly

Beata U. Orzechowska

Daniel E. Otzen

Menno Oudhoff

Toshinori Ozaki

Shogo Ozawa

Petar Ozretić

Chiara Paganini

Jin Ho Paik

Giuseppe Palmieri

J. Palomeque

Maria Paola Germano

Zeev Paroush

William Parson

Basant K. Patel

Jananan Pathmanathan

Theetha L. Pavankumar

Semra Paydas

Michiel Pegtel

Wan‐Xin Peng

Maikel Petrus Peppelenbosch

Shazib Pervaiz

Alessia Peserico

Inés Pinto

Michael Pishvaian

Lilian Irene Plotkin

Damon Poburko

Indira D. Pokkunuri

Stefania Pollastro

Sreenivasan Ponnambalam

Daniil Popov

Doris Popović

Raffaele Porta

Thomas L. Poulos

Chryssa Pourzitaki

David R. Poyner

Monica Prado

Gerrit Johannes Klaus Praefcke

Patricia Prieto

Timothy A. Pritts

Kenneth Pritzker

Peng Pu

Jianmin Qiu

Yunjiang Qiu

Chuan Qiu

Yunjiang Qiu

Chunfeng Qu

Stefania Raimondo

Malini Rajan

Srinu Ratla

Fiona Rawle

Katia Rebola

Miguel Rebollo

Anthony K. Redmond

K.A. Reiss

N. Rhaleb

James Rheinwald

Derek Richard

Karen Rihani

Remy Robert

Sergio R. Roberto

Mark Roberts

Josias Rodrigues

J.L. Rodrigues‐Fernandes

Jenny Carolina Rodríguez

Krysztof Rogowski

Susumu Rokudai

Michael Roleda

Stephane Rolland

Elena V. Romanova

Sandra Romero‐Cordoba

Georcki Ropón‐Palacios

Carlos Ros

Ann K. Rosenthal

Mehryar Habibi Roudkenar

Adhiraj Roy

Yuri Rubtsov

Yuri Rueda

Maja Sabol

Naruhiko Sahara

Pierre Saintigny

Naoya Sakamoto

Valentina Sala

M. Salehi

Madhuja Samaddar

Doaa M. Sami

Luisa María Sandalio

Paola Sanese

Prasanna Kumar Santhekadur

Patricia Santofimi

Marinilce F. Santos

Annalisa Santucci

Luigi Sapio

Devanand Sarkar

Yasushi Sasaki

Naoyuki Sato

Alexander P. Savitsky

Deborah Schechtman

Holger A. Scheidt

Ulf Schmitz

Tony Schountz

Barbara Schreier

Gerrit Schut

Arianna Scuteri

Vincent F. Segers

Masayuki Sekiguchi

Ivana Semova

Serif Senturk

Wongi Seol

C. Sergi

Ivan G. Shabalin

G. Shah

Sally Shalaby

Roshanak Shams

Ping Shao

Bojing Shao

Shun‐li Shen

Yidong Shen

Lei Shi

Hongxue Shi

Eiji Shigetomi

Myounsup Shim

Takahiro Shiotsuki

Toshiro Shirakawa

Arvind Shukla

Séverine Sigoillot

Bartosz Sikora

David Silha

Joao S. Silva

Gregg J. Silverman

N. Silvestris

Fernando Simabuco

Luca Simeoni

Constantine Simintiras

Y. Singh

Izabella Slezak‐Prochazka

Rebecca Slick

Juliana Smetana

Agnieszka Śmieszek

Vytautas Smirnovas

David Smith

Lynne Sneddon

Emilie Snell‐Rood

Luis Felipe Somarribas Patterson

Fangzhou Song

Minseok Song

Lu Song

Oliver Spadiut

Natthachai Srisawat

Madhulika Srivastava

Ashley A. Stegelmeier

Karin Stensjo

Dana Stoian

Richard Strasser

Jeremy Stubblefield

Takayoshi Suganami

Naoto Sugeno

Devi Suman

Wenbing Sun

Bing Sun

Hong Sun

Chang Sun

Zhen Sun

Yao Sun

Wei Sun

Yuan Sun

Subhayan Sur

Katsuhiko Suzuki

Eiji Suzuki

Youri Svitkin

Mansoor Ali Syed

Suchandan Sykder

Piroska E. Szabo

Attila Szanto

Adam Szpechcinski

Carlos Tabraue

Manuel Taft

Ehsan Taghiabadi

Ryo‐U Takahashi

Ryosuke Takahashi

Tetsufumi Takahashi

Hisanori Takenobu

Hideyuki Takeuchi

Masaharu Takigawa

Joanne Tsui Ming Tan

Shaoyuan Tan

Aik Choon Tan

Dale Tang

Shengce Tao

Ran Tao

Umberto Tarantino

Eylem Taskin

Iman Tavassoly

Luba Tchertanov

Gianluca Tedaldi

Felipe RobertiTeixeira

A.J. Teodoro

Jumpei Teramachi

Suman S. Thakur

Anand Thirupathi

Vera Thole

Michael Thorne

Zhifeng Tian

Xuefei Tian

Vinod Tiwari

Vjekoslav Tomaic

Sanna Toppila‐Salmi

Seiji Torii

Vicente A.Torres

Masashi Toyoda

Aleksandar Trifunovski

Chakrapani Tripathi

Marco Tripodi

Angela Trocino

Andres Trostchansky

Jeanie Tryggestad

Kouhei Tsumoto

Smanla Tundup

Kenta Uchibe

Maciej Ugorsku

Philippe G. Urban

Kim Vaiphei

Haritha Vallabhaneni

Mauro Valtieri

Richard van Krieken

Alessandro Vannini

Tunde Varga‐Atkins

Alejandro Velazquez‐Cuz

Mariano Venanzi

Elisa Venturi

Adalberto Ramon Vieyra

Saul Villa‐Trevino

Jaime Villegas

Innokentii E. Vishnyakov

Hans J.F.G. Vliegenthart

Niels Voigt

Hans Von den Hoff

Ivan I. Vorobiev

Nathan Wall

Verena Wally

Shuanglin Wan

Yunguan Wang

Ziyan Wang

Robert Y.L. Wang

Chuanxin Wang

Qunshan Wang

Chen Wang

Ye Wang

Zhicheng Wang

Weili Wang

Xiaoyi Wang

Ziheng Wang

Ting Wang

Zhichao Wang

Zheng‐Fei Wang

Chao‐Yung Wang

Yasuharu Watanabe

Andrew Watson

Keith Webster

Nicholas Webster

Qiushi Wei

Ernesto Weil

Miron Weinreb

Ingrid Weiss

Neale Weitzmann

Yan‐Chen Wen

Meng‐Shih Weng

Johannes Westman

Jacqueline Whatmore

Julian Whitelegge

Lawrence Wilson

Brice Wilson

Daniel N. Wilson

Eytan Wine

Kenneth W. Witwer

Matthew Wohlever

Bartosz Wojtas

David W. Wood

Yuan Seng Wu

Song Wu

Gaosong Wu

Nan Wu

Xue Xiao

Yibin Xu

Zhonghua Xu

Jianping Xu

Xin Xu

Sunil Yadav

Sudhir Kumar Yadav

Murali Yallapu

Kazutaka Yamamoto

Toshimitsu Yamaoka

Zuo‐Qin Yan

Guoyu Yang

Yueh‐Hsun Kevin Yang

Yung‐Hun Yang

Min Yang

Zhaolin Yang

Wan Yang

Yungui Yang

Min Yao

Shinji Yasuhira

Kiyoshi Yasukawa

Adam Ye

Daniel Yee

Jwu‐Lai Yeh

Kelvin Yen

Li Yin

Haidi Yin

Chaoran Yin

Tianlei Ying

Meidan Ying

Masafumi Yohda

Francis Yoshimoto

Atsutoshi Yoshimura

Yingjie Yu

Fenglai Yuan

Yixing Yuchi

Malgorzata Zakrzewska

Andrey A. Zamyatnin Jr

Laura Zannini

Alejandra Zarate‐Potes

Apostolos Zaravinos

Roxanne Zascavage

Alessandra Zecca

Lingyu Zeng

Jinsong Zhang

Daoxiang Zhang

Xian Zhang

Xinyi Zhang

Junjie Zhang

Yi Zhang

Chenlin Zhang

Jian Zhang

Fang‐Jie Zhang

Ye‐Wang Zhang

Yunfeng Zhao

Yongchao Zhao

Wei‐Dong Zhao

Jiajun Zhao

Shushan Zhao

Hui Zhao

Xin‐Chun Zhao

Fei Zheng

Mingjun Zheng

Zhihai Zheng

Mingjun Zheng

Meng Zhou

Feng Zhou

Shengtao Zhou

Yu Jie Zhou

Zhiqun Zhou

Weijie Zhou

Naiqiang Zhu

Yang Zhu

Ziwei Zhang

Zhen Zou

Zhi Zou

Ibrahim Zúñiga‐Chaves

